# MERS-CoV: epidemiology, molecular dynamics, therapeutics, and future challenges

**DOI:** 10.1186/s12941-020-00414-7

**Published:** 2021-01-18

**Authors:** Ali A. Rabaan, Shamsah H. Al-Ahmed, Ranjit Sah, Mohammed A. Alqumber, Shafiul Haque, Shailesh Kumar Patel, Mamta Pathak, Ruchi Tiwari, Mohd. Iqbal Yatoo, Abrar Ul Haq, Muhammad Bilal, Kuldeep Dhama, Alfonso J. Rodriguez-Morales

**Affiliations:** 1 Molecular Diagnostic Laboratory, Johns Hopkins Aramco Healthcare, Dhahran, Saudi Arabia; 2 Specialty Paediatric Medicine, Qatif Central Hospital, Qatif, Saudi Arabia; 3 Tribhuvan University Institute of Medicine, Kathmandu, Nepal; 4 Laboratory Medicine Department, Faculty of Applied Medical Sciences, Albaha University, Albaha, Saudi Arabia; 5 Research and Scientific Studies Unit, College of Nursing & Allied Health Sciences, Jazan University, Jazan, Saudi Arabia; 6 Division of Pathology, ICAR-Indian Veterinary Research Institute, Izatnagar, Bareilly, Uttar Pradesh 243 122 India; 7 Department of Veterinary Microbiology and Immunology, College of Veterinary Sciences, UP Pandit Deen Dayal Upadhayay Pashu Chikitsa Vigyan Vishwavidyalay Evum Go-Anusandhan Sansthan (DUVASU), Mathura, 281001 India; 8 Division of Veterinary Clinical Complex, Faculty of Veterinary Sciences and Animal Husbandry, Sher-E-Kashmir University of Agricultural Sciences and Technology of Kashmir, Shuhama, Alusteng Srinagar, Shalimar, Srinagar, Jammu and Kashmir 190006 India; 9 Division of Clinical Veterinary Medicine Ethics & Jurisprudence, Faculty of Veterinary Sciences and Animal Husbandry, Sher E Kashmir University of Agricultural Sciences and Technology, Kashmir, Shuhama, Srinagar, 190006 India; 10 School of Life Science and Food Engineering, Huaiyin Institute of Technology, Huaian, 223003 China; 11 Public Health and Infection Research Group, Faculty of Health Sciences, Universidad Tecnologica de Pereira, Pereira, Colombia; 12 Grupo de Investigación Biomedicina, Faculty of Medicine, Fundación Universitaria Autónoma de las Americas, Pereira, Risaralda Colombia; 13 School of Medicine, Universidad Privada Franz Tamayo (UNIFRANZ), Cochabamba, Bolivia

**Keywords:** MERS-CoV, SARS-CoV-2, Epidemiology, Molecular dynamics, Phylogeny, Pathology, Therapeutics, Challenges

## Abstract

The Severe Respiratory Syndrome Coronavirus 2 (SARS-CoV-2) has gained research attention worldwide, given the current pandemic. Nevertheless, a previous zoonotic and highly pathogenic coronavirus, the Middle East Respiratory Syndrome coronavirus (MERS-CoV), is still causing concern, especially in Saudi Arabia and neighbour countries. The MERS-CoV has been reported from respiratory samples in more than 27 countries, and around 2500 cases have been reported with an approximate fatality rate of 35%. After its emergence in 2012 intermittent, sporadic cases, nosocomial infections and many community clusters of MERS continued to occur in many countries. Human-to-human transmission resulted in the large outbreaks in Saudi Arabia. The inherent genetic variability among various clads of the MERS-CoV might have probably paved the events of cross-species transmission along with changes in the inter-species and intra-species tropism. The current review is drafted using an extensive review of literature on various databases, selecting of publications irrespective of favouring or opposing, assessing the merit of study, the abstraction of data and analysing data. The genome of MERS-CoV contains around thirty thousand nucleotides having seven predicted open reading frames. Spike (S), envelope (E), membrane (M), and nucleocapsid (N) proteins are the four main structural proteins. The surface located spike protein (S) of betacoronaviruses has been established to be one of the significant factors in their zoonotic transmission through virus-receptor recognition mediation and subsequent initiation of viral infection. Three regions in Saudi Arabia (KSA), Eastern Province, Riyadh and Makkah were affected severely. The epidemic progression had been the highest in 2014 in Makkah and Riyadh and Eastern Province in 2013. With a lurking epidemic scare, there is a crucial need for effective therapeutic and immunological remedies constructed on sound molecular investigations.

## Introduction

The Severe Respiratory Syndrome Coronavirus 2 (SARS-CoV-2) has gained global research attention, given the ongoing pandemic. Nevertheless, a previous zoonotic and highly pathogenic coronavirus, the Middle East Respiratory Syndrome coronavirus (MERS-CoV), is still causing concern, especially in the Kingdom of Saudi Arabia (KSA) and neighbour countries. The prevalence of MERS-CoV across the Middle East region seems to be significant but also have caused imported cases in distant countries. This emerging pathogen is still being reported representing an epidemic threat without effective therapeutics till day [1–4], deserving a thoughtful review of the literature [5–141].

This single-stranded, positive-sense RNA containing virion of the genus Betacoronavirus has been noted from sputum samples in more than 27 countries including the KSA, UAE, Qatar, Austria, Bangladesh, Thailand, Indonesia, UK and USA. Around 2500 cases of MERS-CoV have been reported till now with approximately 35% fatality rate [5–7, 117] since its first detection in the KSA in 2012 [8, 9].

MERS coronavirus was incidentally first reported in the KSA in 2012. The confirmation was done by gene sequencing of the new coronavirus obtained from sputum samples of a patient admitted for suspected flu. Clinical features range from asymptomatic, flu, pneumonia and acute respiratory distress syndrome [118]. In most of the cases, typical infection manifests as lower respiratory tract infection with shortness of breath, cough, and fever, at times, leading to pneumonia that progresses to acute respiratory distress syndrome. The crucial factors for the development of pneumonia include older age, pyrexia, lymphopenia, thrombocytopenia, increment in C-reactive protein in serum (≥ 2 mg/dl) and high viral load in sputum [10, 11]. Depending upon the severity of the disease, respiratory failure, and acute kidney injury are common in patients requiring the extracorporeal membrane oxygenation, ventilation and dialyses [10, 11]. Clinical complications are more severe in MERS than SARS-CoV-2 [119, 120]. Gastrointestinal manifestations, renal and multiple organ failure have been reported amongst fatal cases. With studies pointing towards a zoonotic origin of the virus, especially bats and camels [12, 20], in tandem with numerous reports of atypical human infections, there is sufficient data today, as evidenced by published literature on the dynamics of the viral genome and its phylogeny that can be effectively utilised towards treatment, long-term surveillance and prophylaxis.

The present review highlights MERS-CoV epidemiology worldwide and in the KSA. It describes molecular dynamics, pathology, phylogeny, therapeutics and future challenges that could help devise appropriate prevention and control strategies to counter this important coronavirus.

## Methodology/literature search

The search strategy has been framed effectual by cross-referencing of keywords and successive second stage utility of the references of the articles identified in the first cycle. Once this consortium of studies has been identified, the inclusion criteria were formulated. PubMed and PMC were explored for studies related to MERS-CoV, MERS-CoV prevalence, MERS-CoV origin, molecular genomic dynamics, tissue tropism, pathology, epidemiology, phylogeny, current therapeutics, disease recurrence, mortality and future challenges. The search terms included MERS-CoV seroprevalence, MERS-CoV gene sequencing, molecular dynamics and gene-taxonomic classification, phylogeny, recombination events, tissue tropism of MERS-CoV, treatment and therapeutic trends.

Out of 1040 studies obtained from various sources, 533 were excluded as these were more general on coronaviruses and not about studies on MERS-CoV specifically. Three hundred fifty-three studies were excluded as being comparative studies and not related to specific prevalence, manifestation, genomics, and diagnostic and treatment protocols on MERS-CoV. Editorials [13] were also excluded. Finally, 121 papers relevant to our review were selected. We used a five-step study process for drafting our review. This included searching of research articles on various search engines or databases, screening and selecting of appropriate publications based on inclusion and exclusion criteria, assessing the merit of these papers, data abstraction and data analysis. The selection was completed by following exclusion criteria wherein non-relevant material such as editorials, letters to the editor, and descriptions non-specific to MERS-CoV were excluded. Inclusion criteria wherein specific material were included.

The review summarises the previous studies in an evocative pattern. For assessing quality, two independent experts have evaluated the methodology. The third expert deliberated on variations if any.

The study has been drafted strictly adhering to ‘The Cochrane Reviewers' Handbook’ that provides guidelines regarding healthcare-associated review including clear, discrete focus, properly-outlined objectives, lucidly stating the various cohorts, rationalisations, elucidations, interventions and results [21]. Further, the manuscript has been drafted as per the specificity of the objectives, including the studies providing the relevant data [22]. The EPOC data collection checklist of the Cochrane Effective Practice and Organization of Care Review Group that determines the appropriateness of the methodology for the review has been a valuable guidance tool for this review with apposite attention on non-randomised studies that generate effect estimates indicating the review is more beneficial rather than just compilation [23].

The search period has been from the year 2012 until November 22, 2020. Search information databases, websites and internet search engines employed included Biomed central, Cinahl, Cochrane Library, Embase, Invert, Picarta, PubMed, SCI, www.doh.gov.uk, www.escriber.com, www.google.com, www.nurse-prescriber.co.uk and www.who.org. For inclusion criteria followed were comparison of a research plan, the protocol of groups followed for valuation of post-treatment response and long-term surveillance in MERS-CoV infections, prevention and control, appropriate genomic studies on molecular dynamics, molecular phylogeny, molecular studies on tissue tropism and genomic classification.

## MERS-CoV prevalence in Saudi Arabia and other geographical domains

The report on the first culture of a new coronavirus by Dr. Ali Mohamed Zaki in 2012 [24, 26] from the KSA and the subsequent case in the United Kingdom [27] has triggered a flurry of research on MERS-CoV prevalence especially, the KSA. Three areas of Saudi Arabia, including Eastern Province, Riyadh and Makkah have been found affected severely based on a study involving time-related variation of MERS-CoV from 2012 to 2017 in the Arabian Peninsula [28]. Time-dependent reproductive number (TD-Rs) explored from the data on case counts considering a statistical Auto-Regressive Integrated Moving Average (ARIMA). The epidemic progression in Makkah and Riyadh regions had been the highest in April 2014 and Eastern Province, in May 2013. A statistically significant biannual seasonality has been observed in Riyadh related to the large camel based seasonal-activities [28].

Another study, evaluating time, season, space and spatiotemporal variation of MERS-CoV by Kulldorff’s spatial scan statistics has also identified that the 41.88% of infections occur during the spring season (with seasonal clusters being significant in April and May [28, 29]. The first cases of MERS-CoV were reported from The United Arab Emirates, Qatar, Jordan, Oman and Kuwait however it spread to Riyadh and Eastern Provinces resulted in 80% cases of the region [30–32]. Transmission by travel to Tunisia, the United Kingdom, France, Germany, Korea and Italy was also noted. Secondary transmissions were rare, especially in Germany and Italy [33, 38].

While the bulk of MERS-CoV cases reported have been related to hospital infections, frequent occurrence of MERS is due to community spread, while as camels are the intermediate hosts. Studies have established that about 50% of camel workers in the KSA have been previously infected as evidenced by MERS-CoV-specific enzyme-linked immunosorbent assay (ELISA), immunofluorescence assay (IFA), neutralising antibody titers and T cell responses. Thus, there is a continuous transmission of MERS-COV from camels to camel workers showing mild disease or remaining asymptomatic, thus infecting healthy persons and the infection spreads further by nosocomial route principally through healthcare workers [39–44]. MERS-CoV can survive in camel milk for more prolonged periods; hence camels are considered as intermediate hosts when the original hosts speculated to be bats is not yet clear [119, 121]. It is still under investigation whether MERS-CoV is transmitted through direct or indirect contact, airborne droplets or ingestion [122].

Severity and mortality due to infection conceivably seem to be dependent on the host immune status and various factors such as comorbidity [45–48]. A recent retrospective study on MERS-CoV related mortality evaluated by the clinical predictors has also established age, total white blood count, neutrophil percentage, serum albumin concentration, utilisation of continuous renal replacement therapy (CRRT) and corticosteroids as potential predictors. CRRT and corticosteroid use has shown the highest odds ratio of 4.95 and 3.85, respectively [49]. Delay in implementation of preventive and control measures are considered as main reasons for the rise in MERS cases in KSA especially in health workers and those in close contact with camels despite the ample availability of effective conventional and advanced molecular diagnostics like reverse transcriptase real-time polymerase chain reaction (RT-qPCR) [49–56]. People to people transmission in MERS is due to jumbling in emergency wards of hospitals or waiting rooms, inadequate hygienic facilities such as handwashing [57, 94]. Less than 50% of infected cases can transmit infection in other people with whom they come in contact when the close contact and negligence, especially in hospitals and households can increase risk of spread [123].

Statistical data in the KSA also point to a high level of uncertainty and knowledge gap among healthcare workers as a cause of increased concern. These include issues related to individual and institution, epidemiological investigations parameters including surveillance, quality data reporting, readiness and competence for implementing measures and responding to MERS-CoV outbreaks [58]. Quick collection of samples by standardised protocols, analysis and dissemination of epidemiological information during infectious disease outbreaks are crucial for filling in these knowledge gaps [59]. Diagnosis by PCR is considered as best option however those with negative PCR tests can be evaluated by serological tests including serum neutralisation assays [124], microarrays [125], and ELISA or micro-neutralisation test [126].

## Phylogenetic scrutiny

The molecular dynamics of MERS-CoV, a positive-sense, single-stranded RNA virus of the genus Betacoronavirus has been perhaps studied extensively not only due to the morbidity and mortality rate of the viral infection but also due to its similarity with an already existing array of known strains of pathogenic human coronaviruses that cause respiratory distress syndrome, viz., HCoV-229E, HCoV-NL63, HCoV-HKU1, HCoV-OC43 and SARS-HCoV [60–62].

Earlier molecular studies have established MERS-CoV as a novel member of the Betacoronavirus, lineage C and the genome, phylogenetically divided into clades, A and B; the earliest cases of MERS being clade A clusters (EMC/2012 and Jordan-N3/2012), and later cases being genetically distinct were put under clade B. With most of the molecular studies falling between 2012 and 2015, 182 genomes have been sequenced (94 have been from human beings and 88 from dromedary camels) and all the sequences have been shown to have more than 99% similarity [62, 63, 67].

Investigations indicate that the MERS-CoV originated around 2011 with pieces of evidence of existence in Central and East Africa, especially in two species of bats, dromedary camel, and European hedgehog as natural hosts [64]. Besides, MERS-CoV is reported to be endemic in bats and camels along with subsequent spread to humans [65, 66].

Research studies have established that recombination has been shared among the members of betacoronavirus [67] and such recombination events have been responsible for the creation of new viral strains capable of infecting new hosts surpassing their immune system. The SARS-CoV virus was opted for many mutations in civets before spillover to humans. Similarly, the MERS-CoV reported to undergo many recombination events and circulated for around 30 years in dromedaries before the outbreak [68, 69]. However, dromedaries imported from the Arabian peninsula to Australia in the last century do not contain MERS. It is, perhaps means that spillover from bats to dromedaries has happened later.

Moreover, after jumping species barriers exogenous viruses before arrival were reported to opt for adaptation in the diverse environment and different hosts [70]. Amongst the two MERS-CoV clades, A and B, there is evidence that shows that the recombination events could have most probably happened amid group III and group V [62, 71]. The recombination events between the other groups and between the clads are yet to be established clearly.

Of the 74 whole-genome sequences of MERS-CoV studied from 9 countries, human and camel isolates formed one cluster and bat/hedgehog isolates formed a primary paraphyletic group to all camel and human MERS-CoV clade. Further investigations have established that the MERS-CoV isolate GI: 589,588,051 obtained from a camel in Egypt represents the first or primary clade to human and the other camel MERS-CoVs. Analysing the whole-length genome sequences of MERS-CoV it has been found that 28 have undergone potential recombination events including 3 of camel and 25 of human MERS-CoVs [62, 72]. This study also revealed the common origin of the entire outbreak in Saudi Arabia [72].

An adaptive evolutionary investigation in the same cohort to determine the selection burden on the MERS-CoV structural proteins during cross-infection has established that except a prevalence of a strong positive selection (with nine prospective selection sites) in spike (S) glycoprotein there has been no such selection in other genes of MERS-CoV [62, 73].

Full genome analysis of an African bat virus which is closely related to MERS-CoV and shows that human, camel, and bat viruses belong to the same viral species [127]. It also indicates MERS-CoV originated in camels and transmitted to humans, not vice versa. MERS-CoV emerged due to exchanges of genetic elements between different viral ancestors which might have taken place either in bat ancestors or in camels acting as mixing vessels for viruses from different hosts [127].

## Molecular dynamics and molecular pathology

The genomic profile of MERS-CoV is over thirty thousand nucleotides in length, with seven predicted open reading frames (ORFs) (ORF1a, ORF1b, ORF3, ORF4a, ORF4b, ORF5 and ORF8b) and four structural genes (S, E, M, N) [74–76]. The two ORFs (ORF1a, ORF1b) encodes replicase complex whereas remaining five accessory ORFs encodes five accessory proteins which play a crucial role in the infection and pathogenesis [77]. The four structural genes viz., S, E, M, N encodes spike, envelope, membrane and nucleocapsid protein, respectively [74, 75]. Spike protein is located on the surface. It has been established to be one of the significant factors in their zoonotic transmission through virus-receptor recognition mediation and subsequent initiation of viral infection. The S protein of MERS-CoV is a transmembrane protein having two subunits S1 and S2. The S1 subunit has a receptor-binding domain (RBD) that binds with dipeptidyl peptidase 4 (DPP4) receptor of the host [11, 78]. MERS-CoV utilises cellular DPP4 receptor of the host for cell entry through binding of its S protein [79]. The main membrane fusion unit is formed by heptad repeats H1 and H2 of S2 subunit [80]. The envelope (E) protein has its role in assembly, intracellular transport and budding of MERS-CoV [81] whereas the membrane (M) protein is required for viral assembly and morphogenesis [82]. All four structural proteins viz. N, S, E and M proteins interact together to form a complete virus particle [83]. Binding of S protein of MERS-CoV to the host cellular receptors results in attachment and start of an infection. This is to follow by fusion of viral envelope with host cell membrane triggered by cleavage of S proteins facilitated by cellular proteases. Hence the availability of these cellular proteases after receptor attachment is considered as the main step determining viral entry [84].

Further, specific mutations in a receptor-binding domain on N terminal of the S protein have been shown to determine its cross-species infection capabilities [55, 56, 85]. Similar four amino acid substitutions have further substantiated this fact in the S protein receptor-binding domain in SARS-CoV and two amino acid substitutions in HKU4 that determine the host infective capability of these viruses respectively. Studies have also established the role of heptad repeat regions in C-terminal of MERS-CoV and related coronaviruses in cross-species transmission [55, 56, 86]. Earlier studies on MERS have established that similar to other corona viral infections, two non-structural polyproteins (pp1a and pp1ab) of MERS-CoV are synthesised in the host cells and then cleaved (a vital step in viral maturation process) by two coronaviral proteases, the main protease Mpro and a papain-like protease [87]. The MERS-CoV Mpro, namely nsp5 of the pp1a proteins (residue 3248–3553), has been elucidated containing a catalytic dyad consisting of a His and a Cys residue [62, 88]. Moreover, crystallographic studies on the structure of MERS-CoV Mpro have shown that it has a scaffold related to that of other coronaviral Mpros with chymotrypsin-like domains I and II and a helical domain III consisting of 5 helices. Investigation through ultracentrifugation has further revealed that MERS-CoV Mpro goes through a process of conversion where it turns from a monomer to dimer involving a peptide substrate and Glu169 is essential for dimerisation and catalysis [62, 89].

Recent studies have established that nsp1 of this coronavirus has an endonucleolytic RNA cleavage function. This helps in the generation of infectious virus particles in specific human cell lines by suppressing host gene expression in infected cells via translation inhibition and endonucleolytic cleavage induction of host mRNAs. The evaluation of wild-type has further confirmed this translation inhibition and endonucleolytic RNA cleavage induction function of the nsp1 and its importance in viral replication and two mutant types MERS-CoV that either lacked one or both of the attributes. Vero cell replication has also established similar results with the wild-type degrading mRNA and inhibiting translation in the host indicating that nsp1 suppresses expression of the host genes. The study suggests that MERS-CoV nsp1 is the first coronavirus gene 1 protein which has an active role in virus multiplication whose RNA cleavage-inducing function has been demonstrated [62, 90].

Studies on the NF-κB signalling mechanism stimulated by (E) protein of coronaviruses is worth the mention in this context. Numerous chemical inhibitors of NF-κB signalling have been shown to reduce lung pathology and inflammation. The E protein inhibits the cellular stress response of the host besides apoptosis. The E protein has been found to have ionic channel activity that could disrupt permeability of the blood vessels and thus can cause fluid exudation in the pulmonary tissues on an infection. Thus, the virus seems to encode an array of genes which affect the natural immunity of the people [62, 91].

Studies on an IFN-stimulated gene (ISG) expression in Calu-3 human respiratory epithelial cells has further established the capability of MERS-CoV to hide from the host immune system. The ISG transcripts and proteins have been found only after an established MERS-CoV infection with peak titres after about 24 h. Further, a significantly decreased expression of ISGs and down-regulation has been reported with MERS-CoV. There may be some other molecular mechanism operating for ISGs expression as gene expression was not downregulated [62, 91, 92]. Moreover, cells infected with MERS-CoV have affected chromatin structures which result in the inability of transcription factors to reach and bind with some ISG promoter regions. The mechanism for this alteration is still under investigation; however, it is suggested that an epigenetic mechanism may be involved in alteration of the structure of chromatin, disrupting the expression of genes in the host [93].

**Fig. 1:**
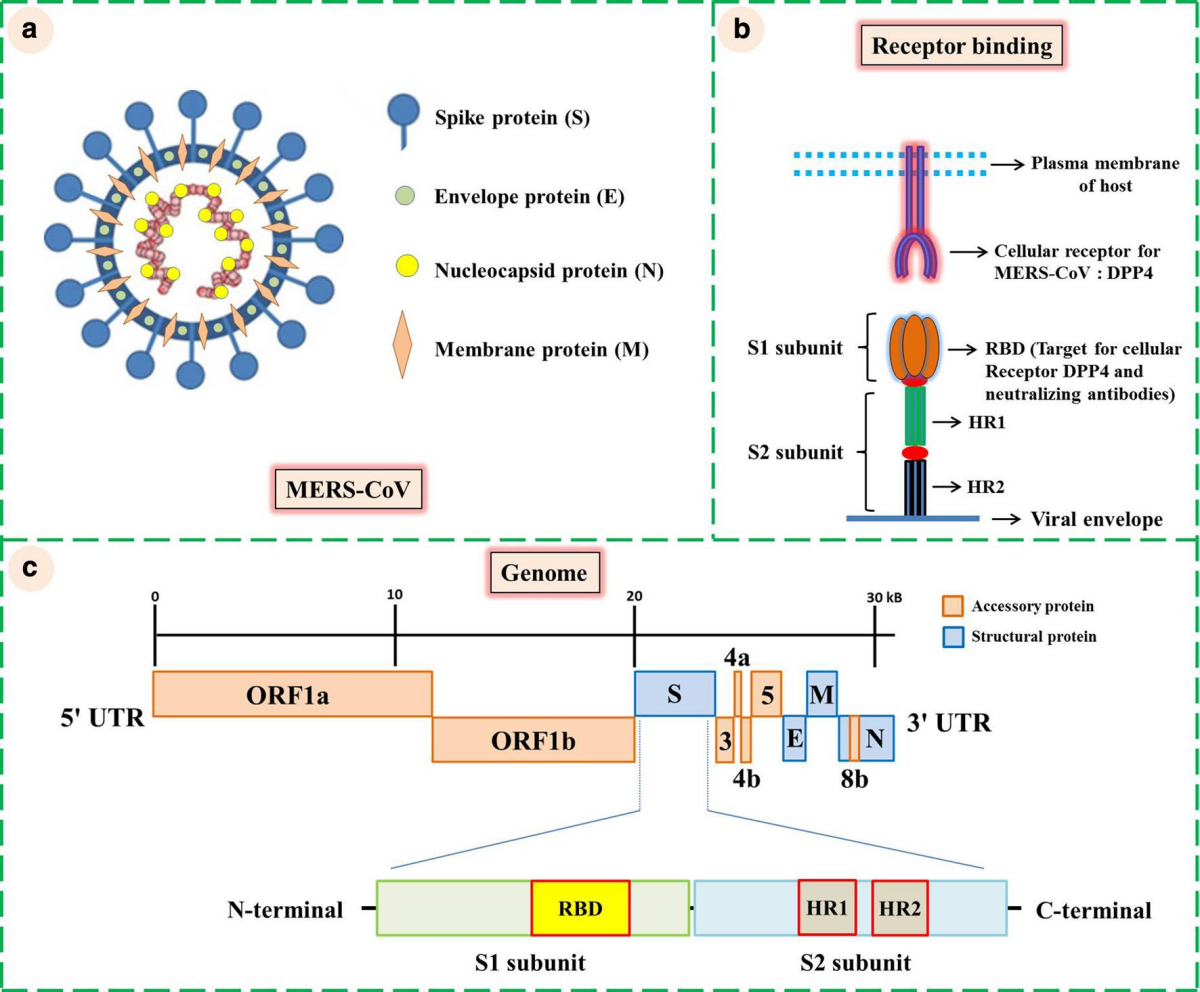
Schematic structures of MERS-CoV proteins. **a** Structure of MERS-CoV along with structural proteins; **b** structure of MERS-CoV spike protein and mechanism of receptor binding; **c** genomic organisation of MERS-CoV

Genomic organisation of MERS-CoV and schematic structures of its proteins is presented in Fig. 1.

## Tissue tropism with therapeutic implications

Molecular studies using single-particle cryo-electron microscopy have established that the spike (S) protein is the principal cause of tissue tropism of this coronavirus. Trimers of these surface S proteins of the virus facilitate the viral binding to the surface receptor of the host cell and subsequent merging of the membranes of the virus and host cell. Adaptation to receptors, changes in the stimulation of proteolytic cleavage and variation in metastability of S protein have been identified as potential factors that determine the tissue tropism [55, 94].

More recent studies have elucidated that virus utilises the S1B domain of the S protein for binding to its functional receptor dipeptidyl peptidase 4 (DPP4), and its S1A domain for binding to sialic acids. Further, the presence of DPP4 in humans, and various animal species including bats, camelids and pigs have been shown to correlate with MERS-CoV host tropism, underlining the importance of DPP4 in viral pathogenesis and transmission [46, 95].

These recent studies have further established that there are variations in the binding of MERS-CoV S1A to sialic acid residues, tissues and host species. There are species-specific differences in the binding of MERS-CoV S1A to the α2,3-sialic acids on host cells, and it does not bind to all the sialic acids on all the tissues. Using nanoparticle-based approaches it has been seen that the virus nanoparticle binds specifically to sialic acid residues present on nasal epithelial tissue of single-humped camel, type II pneumocytes present in the lungs of humans, and enterocytes of typical pipistrelle bats. The S1A nanoparticles, on the contrary, had no bonding with the intestinal epithelium of bats, the nasal epithelial tissue of swine and rabbits. Thus, these specific bindings of S1A domain to the nasal, respiratory and intestinal epithelium of dromedary camels, humans and typical pipistrelle bats, respectively suggests the significance of this domain in infection and tissue tropism [47, 55, 97].

Molecular investigations have revealed that spike envelope protein S is composed of 20-nm-long homotrimers and the N-terminal subunit of each S protomer, called S1, is folded into four discrete dominions labelled S1A to S1D. Studies have further established that the viral binding to host cells is mediated through S1B dominion of the virus and dipeptidyl peptidase 4 (DPP4) receptor of the host. DPP4 receptor is less expressed in the nasal mucosa and epithelium of the upper airways but more abundant on the epithelium of distal airways along with type I and II pneumocytes. Additionally, DPP4 receptors were highly expressed on non-ciliated cells of bronchial epithelium, cells of endothelium and few haemopoietic cells along with epithelium of other organs like liver, kidneys, thymus, bone marrow and intestine suggesting the possibility of widespread dissemination of the infection in the body [79, 98]. Apart from DPP4 receptor, the virus, as evidenced by previous studies has also been shown to bind to sialic acid of the respiratory epithelium of camels and humans including both upper and lower tracts, via S1A. Thus, it is suggested that viral-host binding can be impeded by modifications in sialic acid that includes 5-*N*-glycosylation; 9-*O*-acetylation and reduction of cell surface sialic acid by application of neuraminidase during the pre-attachment or early attachment phase [99].

Earlier computational immunological studies employing different in silico tools and Immune Epitope Database (IEDB) on the RBD of S glycoprotein of MERS-CoV have identified the antigenic epitopes of the virus. Of these 8 T-cell epitopes are found to be potential candidates, and 19 are major histocompatibility complex (MHC) class-I alleles based on molecular docking investigation using specific HLA allele for elucidating binding affinity. These epitopes with maximum interaction and antigenicity can be explored as vaccine candidates. The epitope, CYSSLILDY, has been shown to have demonstrated interactions with many MHC -I molecules besides adequate B-cell antigenicity hence can be explored as subunit vaccine for MERS [52, 100]. More recent molecular studies have elucidated an ORF1ab sequence that encodes replicase polyproteins has significance in MERS-CoV infection process. Hence, ORF1ab replicase polyprotein has been suggested as a viable candidate for effective MERS control [46, 101].

Control of viral activity by RNA interference (RNAi) technology is yet another breakthrough study. It helps in the silencing of genes after transcription with a particular sequence. Variation of the genome of different isolates of virus posses a great difficulty in proposing miRNAs and siRNAs that can interfere with candidate genes. One such study has constructed 4 potent miRNA and 5 siRNA molecules that would silence 9 MERS-CoV strains with sufficient difference in nature leading to impeded viral infectivity. The siRNA and miRNA molecules fabricated for ORF1ab gene of various strains of MERS-CoV provides a potential avenue for the laboratory synthesis of antiviral RNA molecules at genomic plane [46, 101]. Temporins are the smallest antimicrobial peptides (AMPs) with antimicrobial immunity effects and may be developed into therapeutic targets against MERS-CoV after thorough in vivo and in vitro studies [102].

## Treatment of MERS

At present specific treatment for MERS-CoV is lacking, but several therapeutic options targeting various elements of the virus are currently under development or available [128]. The various therapeutic strategies used in severe cases of MERS include immunotherapy by convalescent plasma and intravenous immunoglobulins, antiviral agents such as protease inhibitors, interferons, ribavirin, alisporivir and their combination along with corticosteroids [129–132]. Absence of precise data on specific treatment prioritised the need for controlled trials [103]. One such trial on convalescent plasma or hyperimmune IV immunoglobulin (HVIG) for the treatment of MERS cases was started in 2014 for determining its safety and efficacy [104] based on the results from SARS and influenza cohort. Moreover, as many as 41 clinical trials for establishing the efficacy of various drugs and vaccines are already launched, and results are expected soon (clinicaltrials.gov). The preclinical studies on the use of convalescent serum from immune camels to infected mice revealed weight loss. They reduced lung pathology [105] suggesting its therapeutic potential and need of the further clinical trial. As per a report, the use of convalescent plasma (PRNT titre of 1/80 or more) in MERS patients with respiratory failure resulted in neutralising activity and thus concludes its efficacy [98, 106]. Monoclonal and polyclonal neutralising antibodies like novel chimeric camel and human heavy chain antibodies were reported to be protective in different animal models and may prove crucial in outbreak management [107–109].

Several agents like interferons, ribavirin, cyclosporine and mycophenolic acid showed inhibitory effects against MERS-CoV in cell cultures [112–115]. Among antiviral agents, high doses of ribavirin have shown significant anti-MERS-CoV activity in-vitro and have been used to manage MERS patients in Saudi Arabia and France [129]. Moreover, ritonavir and lopinavir combination has shown efficacy against MERS-CoV in-vitro. In this context, the FDA has extended the indications of lopinavir for MERS-CoV. Besides, two case reports from Greece and Korea have described improvement in MERS patients after treatment with lopinavir, ribavirin and type 1 interferon [130]. About this, a phase II-III clinical trial is launched to study and evaluate the efficacy, safety and feasibility of the lopinavir/ritonavir/recombinant IFNβ-1b combination vs placebo in MERS patients (clinicaltrials.gov). In a study, use of IFN-α-2a and ribavirin has been reported to have a potent inhibitory effect on the MERS-CoV replication along with improvement in patient survival [133]. Alisporivir is a cyclophilin inhibitor and reported to provide in-vitro anti-MERS-CoV activity when used along with ribavirin; however, in-vivo studies supporting its use are lacking [131].

However, infection with MERS-CoV reduces the response of host for interferon, MERS-CoV is reported 100 times more sensitive to IFN-α treatment. Hence, several retrospective cohort studies have been conducted using IFN-α in combination with lopinavir, ribavirin or mycophenolate mofetil (MMF) to establish a treatment regimen for MERS. Although none of the studies has reported increased overall survival, one study reported increased survival in the case of critically ill intubated and ventilated MERS patients [134]. Moreover, a combination of lopinavir-ritonavir, pegylated interferon alfa-2a and ribavirin has been used in severe cases of MERS. Besides, a randomised clinical trial comparing lopinavir-ritonavir and interferon-beta 1b with supportive care against supportive care and placebo are in progress in Saudi Arabia to know the efficacy of this combination therapy regimen in severely affected patients [98]. The results of the study reported that a combination of recombinant interferon beta-1b and lopinavir-ritonavir resulted in lower mortality than placebo among laboratory-confirmed MERS patients. Besides, the most significant effect was observed when treatment was started within 7 days after the onset of symptoms [132].

Chloroquine and nitazoxanide are reported to have anti-MERS-CoV activity in-vitro, and the FDA has already approved the chloroquine for treatment of MERS. In contrast to this, no clinical data or studies support its in vivo use at present. In vitro study demonstrated two daily oral doses of nitazoxanide. No clinical data or studies support its use in vivo at present [135].

Mycophenolate mofetil (MMF) is an immunosuppressant and reported to have anti-MERS-CoV activity when administered in adequate doses in humans. Moreover, MMF seems to have a synergistic effect with IFN-β1b in-vitro when used for the treatment of MERS [136]. In contrast to this, non-human primate (common marmosets model), treated with MMF, reported developing more severe lesions and higher case fatality rate in comparison to untreated animals [136]. In contrast with the animal model, the IFN-β1b/MMF combination was found beneficial in the treatment of MERS in Saudi Arabia [137]. Besides, silvestrol, a molecule found in plants of flavagline family reported to bind with eIF4A and in turn enhances the affinity of eIF4A for mRNA. That subsequently blocks the helicase activity and inhibits protein translation. Concerning this, a recent in-vitro study demonstrated the anti-MERS-CoV activity of the silvestrol [138], but in-vivo studies are no conducted till now to establish the same [139].

Recently, a retrospective study was carried out to establish the use of extracorporeal membrane oxygenation (ECMO) as salvage treatment for critically ill MERS patients with respiratory failure in Saudi Arabia [140]. The study included MERS patients from five ICUs from 2014 to 2015 and consisted of two groups viz ECMO versus conventional treatment. A total of 35 patients with similar baseline characteristics were included in the study, among which 17 were treated with ECMO against 18 who received conventional care. The results of the study supported the use of ECMO for MERS patients with respiratory failure as salvage treatment [140].

A retrospective study revealed treatment of severely ill MERS patients with macrolides not resulted in the reduction of mortality and accelerated clearance of MERS-CoV RNA in comparison to non-treated patients [110] suggesting that antibiotic therapy may only be used to control secondary bacterial infection with no change in virus-associated outcome. Consistent efforts for identifying specific and effective therapeutics or prophylactics have, of course, brought about research breakthroughs. The disruption of accessory ORFs may provide a crucial platform for the attenuation of emergent strains of MERS-CoV soon.

Additionally, for therapeutics and vaccine development against MERS-CoV and related coronaviruses, the accessory ORF functions may be targeted [76, 77]. The broad-spectrum antiviral nitazoxanide showing in vitro activity MERS-CoV; the GLS-5300, which is an aDNA-plasmid vaccine that codes S protein, and the monoclonal antibody, m336 are quite promising [46, 111]. A study reported that patients showed anger and symptoms of anxiety when quarantined hence accurate information, an appropriate supply of food and clothes along with mental health support is utmost necessary to isolated individuals [116]. Until appropriate therapeutics or prophylactics are developed prevention by awareness and education is vital for preventing future outbreaks [141].

**Fig. 2:**
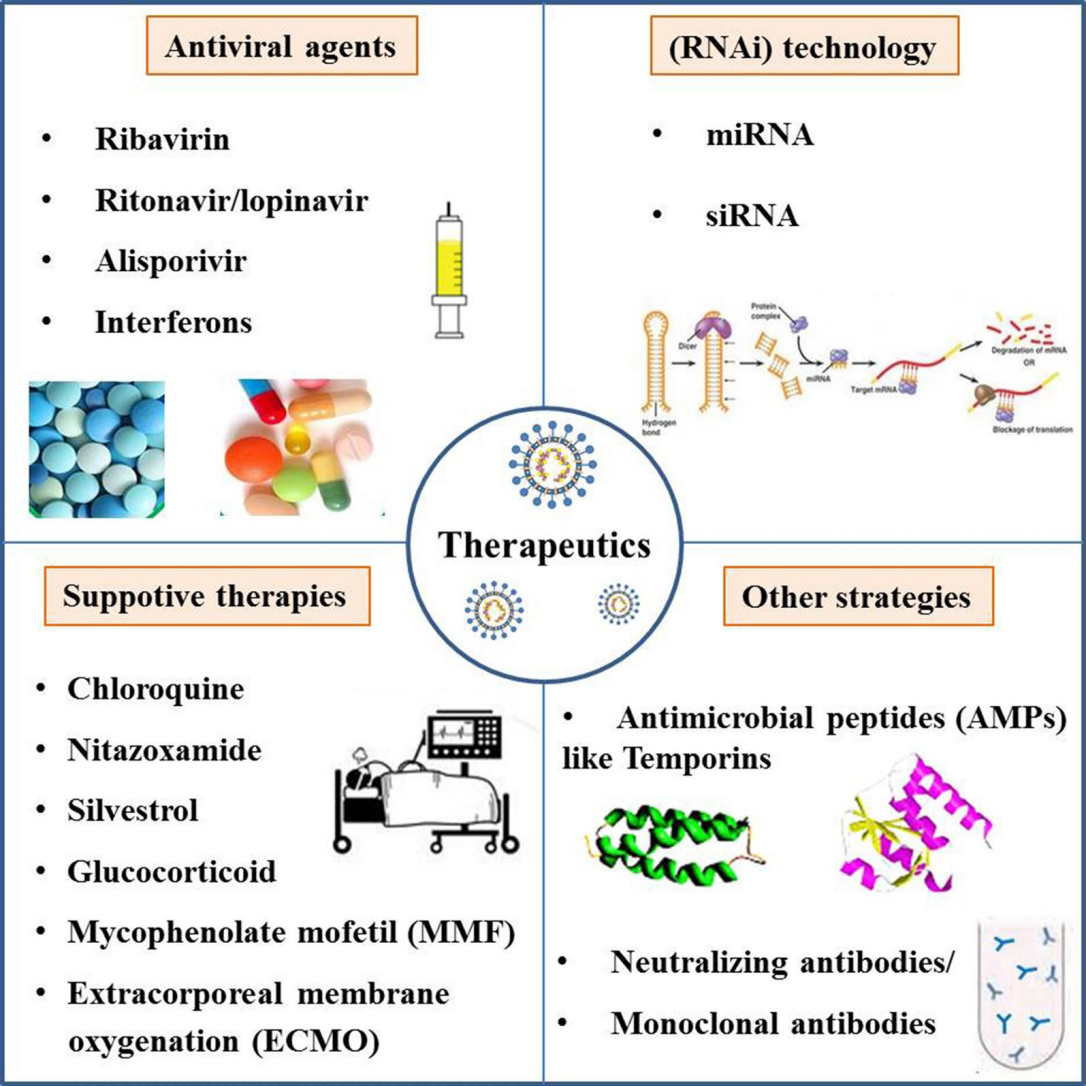
Therapeutic strategies available against MERS

Therapeutic strategies against MERS-CoV are presented in Fig. 2.

## Conclusion and future prospects

The MERS-CoV possesses the epidemic potential and continues to occur in the form of sporadic disease of humans, which remains on the Blueprint 2020 priority list of WHO, in addition to SARS-CoV, SARS-CoV-2 [142] and other emerging and life-threatening pathogens. MERS-CoV infection poses a serious health risk not only in the KSA but across the continents due to its zoonotic community acquisition and rapid nosocomial transmission [143]. The MERS-CoV is reported to be highly endemic among camels from broad areas of Africa and the Middle East, including Saudi Arabia. Moreover, the possibilities of zoonotic transmission with a significant risk of human epidemics are most likely to continue for years shortly [144]. With an established sequence of recombination events amongst the members of this beta-coronavirus within a short period, the prevalence of new viral strains capable of infecting new hosts surpassing their immune system is but reality soon [145].

Further, the inherent genetic variability amongst various clads of the MERS-CoV paves the way for an inevitable new cross-species jumping process of these enveloped, plus-stranded RNA viruses with inter- and intra-species tropism changes. Real-time reverse-transcription-polymerase-chain-reaction (RT-PCR) is essential for the diagnosis of MERS-CoV from respiratory secretions; however, there is no efficient antiviral therapy yet. Since studies have shown that glucocorticoid therapy is related to higher mortality, the treatment remains mostly supportive. Recently, a diagnostic detecting the region upstream of the envelope gene (upE) and ORF1a have also been in use for screening and laboratory confirmation.

Investigative studies on the antigenic epitopes of the viral spike (S) entry protein, host dipeptidyl peptidase 4, and sialic acid-binding, ORF1ab sequencing, transcriptional gene silencing utilising RNA interference (RNAi) technology are, no doubt, noteworthy recent discoveries from a therapeutic and prophylactic perspective. With a lurking epidemic scare in the immediate future and the higher (43%) case fatality ratio of MERS-CoV compared to that of SARS-CoV (11%) and SARS-CoV-2 (approximately 10%), there is a crucial need for effective therapeutic and immunological remedies constructed on sound molecular investigations. Keeping the great epidemic potential of MERS-CoV in consideration countries at risk must invest a robust amount of funds in surveillance, monitoring, public health research [146] and healthcare infrastructure along with vaccine development for human and camel. After SARS-CoV-2, other coronaviruses epidemic may overcome in the Middle East and the Word, including the possibility of re-emergence of MERS-CoV in new areas, as well as its persistence in the region, deserving more research and attention as has been stated by the World Health Organization.

## Data Availability

Not applicable.
